# A gene-specific variance-control approach corrects polygenicity-driven inflation observed in transcriptome-wide association studies

**DOI:** 10.1016/j.ajhg.2025.12.014

**Published:** 2026-01-20

**Authors:** Yanyu Liang, Festus Nyasimi, Hae Kyung Im

**Affiliations:** 1 Section of Genetic Medicine, University of Chicago, Chicago, IL, USA; 2 Computing Environment and Life Sciences Directorate, Argonne National Laboratory, Argonne, IL, USA

**Keywords:** transcriptome-wide association studies, TWASs, type I error inflation, polygenicity, heritability, variance control

## Abstract

Transcriptome-wide association studies (TWASs) and related methods (xWASs) have been widely adopted in genetic studies to understand molecular traits as mediators between genetic variation and disease. However, the effect of polygenicity on the validity of these mediator-trait association tests has largely been overlooked. Given the widespread polygenicity of complex traits, it is necessary to assess the accuracy of these mediator-trait association tests. We found that, for highly polygenic target traits, the standard test based on linear regression is inflated, leading to dramatically increased false-positive rates that grow linearly with sample size and heritability. To address this inflation, we propose an effective variance-control method—similar to genomic control but allowing for a different correction factor for each gene. Using simulated and real data, as well as theoretical derivations, we show that our method yields calibrated false-positive rates, outperforming existing approaches. We further demonstrate that methods analogous to TWASs, namely those that associate genetic predictors of mediating traits with target traits, suffer from similar inflation issues. We advise developers of genetic predictors for molecular traits (including polygenic risk scores, PRSs) to compute and provide the necessary inflation parameters to ensure proper false-positive control. Finally, we have updated our PrediXcan software package and resources to facilitate this correction for end users.

## Teaser

We show that transcriptome-wide association studies (TWAS) and related methods (xWAS) using genetic predictors suffer from inflated false-positive rates for highly polygenic complex traits, increasing linearly with sample size and heritability. We propose a variance-control method that yields well-calibrated results when applied to both simulated and real data.

## Introduction

To explain the mechanisms behind the hundreds of thousands of loci discovered via genome-wide association studies (GWASs), researchers have studied the role of molecular traits as mediators. Transcriptome-wide association studies (TWASs) and related molecular-mediated-trait association methods (referred to here as xWASs) perform association tests between genetic components of molecular traits and the target trait, evaluating one feature at a time.^1^^,^^2^^,^^3^ Molecular pleiotropy (i.e., when the same variant affects the expression of multiple genes but only one gene influences the target trait) and linkage disequilibrium (LD) contamination (i.e., when the variant associated with the molecular trait has no effect on the trait but is in LD with a trait-altering variant) are known to increase the false-positive rate of xWAS methods.^4^^,^^5^^,^^6^^,^^7^^,^^8^ Despite these limitations, xWAS methods—including cistrome-wide association study (CWAS), regulome-wide association study (RWAS), proteome-wide association study (PWAS), and isoform-level TWAS (isoTWAS)—are widely acknowledged as useful for nominating molecular traits driving the etiology of complex traits.^3^^,^^9^^,^^10^^,^^11^

Some prior studies have reported potential inflation of false positives in TWASs. For example, van Iterson et al.^12^ argued that TWAS results tend to be biased and inflated, as indicated by deviations from the expected null distribution. The authors concluded that the standard genomic control method overcorrected for this observed inflation and proposed a Bayesian approach termed BACON to estimate the empirical null distribution as a solution. However, their assumption that most features are not be associated with the target trait may be invalid due to the broad polygenicity of complex traits.^13^ We demonstrate that BACON’s genomic-control approach does not fully resolve the inflation caused by the target trait’s polygenicity.

de Leeuw et al. also suggested that TWASs may produce inflated type I error, attributing this inflation to inaccuracies in predicting gene-expression traits.^14^ However, error-in-variables theory^15^ assures us that, although noisy predictors reduce the power of the association, they do not cause an inflation of type I error as long as the prediction error itself is not associated with the outcome. In line with this, our findings indicate that inflation reported in TWAS/xWAS results is better explained by the polygenicity of the target trait than by prediction error.

It is increasingly accepted that there is widespread polygenicity of most complex traits.^13^^,^^16^^,^^17^ Indeed, the effect of polygenicity has been explored and leveraged in the context of GWAS with methods such as LD score regression (LDSC) and related approaches.^18^ However, while the overall genetic contribution of xWAS predictions to complex traits has been characterized,^19^^,^^20^ the effect of polygenicity on xWAS and the inflation of results has not been investigated rigorously.

In this study, we show that, even in the absence of known false positives caused by molecular pleiotropy and LD contamination, the widespread polygenicity of the target trait leads to inflation of the association statistic. In other words, the false-positive rate is higher than estimated by standard methods. To maintain the utility of xWAS methods, it is essential to ensure that false-positive rates are well calibrated.

We begin by demonstrating that polygenicity induces inflated type I error, even in a simple setting where SNPs are independent of one other and the error terms. Next, we show this inflation with real genotype data by assessing the association between genetically predicted expression in the UK Biobank and polygenic null traits—simulated polygenic phenotypes that have no causal relationship with the predicted expression. We further show that this inflation is not limited to a specific software but is a broader property of the TWAS/xWAS approach. We show inflation increases linearly with GWAS sample size and the heritability of the target phenotype, consistent with real TWAS results for 110 GWAS traits as well as our theoretical derivations. Finally, we propose a user-friendly correction strategy—termed variance control—and demonstrate its effectiveness using both null and actual GWAS traits.

## Methods

TWAS and related methods seek to identify potential causal mediators (e.g., gene expression, protein levels) by testing the effect of the mediating trait on a target trait. We describe the model and usual assumptions here.

$$Y=T\beta +\epsilon_{\text{twas}}$$

(Equation 1) $$T=\sum_{k}X_{k}\cdot \gamma_{k}\text{,}$$

where *β* is the (fixed) effect of the mediating trait (*T*) on the target trait (*Y*), and *ϵ*_twas_ is the error term independent of the mediator (*T*). Only the genetic component of the mediator can be tested, so we define *T* as a linear combination of genotype dosages. Genetic effects on the mediator are defined as *γ*_*k*_ and genotype dosages as *X*_*k*_, where *k* indexes the genetic variants. This model accommodates both sparse (most *γ*_*k*_ = 0) and polygenic architecture (most *γ*_*k*_ ≠ 0) for the mediating trait.

The standard TWAS approach assumes that genetic variants affect the target trait only through the mediating trait (i.e., no horizontal pleiotropy). When the target trait is not polygenic, this assumption is more likely to hold and the TWAS test statistic typically follows the expected null distribution, yielding well-calibrated type I error rates. However, when the target trait is polygenic, horizontal pleiotropy becomes more probable, leading to inflated false-positive rates as we demonstrate below.

To evaluate the TWAS approach where this assumption is satisfied, we defined the null trait as a simulated phenotype that has no causal relationship with any gene or other mediating molecular trait. The null trait may or may not have a polygenic background, which we refer to as a polygenic null trait or a non-polygenic null trait, respectively.

### Simulation of minimal example illustrating inflation

We tested the calibration of TWASs under ideal conditions (i.e., no horizontal pleiotropy) by conducting simulations using a simple setting based on Equation 1 for target traits with and without a polygenic background. We call this minimal example to indicate the simplest example where we can find inflation. We ran association tests under the null hypothesis, setting *β* = 0 for all simulations.

### Simulate genotype and mediating trait

We simulated genotype data for 1,000 individuals (*N*) and 999 independent SNPs (*M*), with a minor allele frequency (MAF) of 0.4, using a binomial distribution (i.e., assuming Hardy-Weinberg equilibrium). SNP effect sizes (*γ*_*k*_) are drawn from a normal distribution, representing genetic effects on the mediating trait. The mediating trait is computed as $T=\sum_{k}X_{k}\cdot \gamma_{k}$, where *X*_*k*_ represents the *k*th column of the *N* × *M* genotype matrix *X* and *γ*_*k*_ represents the vector of *M* SNP effects on *T*.

### Non-polygenic null trait simulation

In this scenario, we assumed that SNPs have no direct effects on the target trait (*Y*). The target trait (*Y*) is simulated by sampling *Y* = *ϵ*_twas_ from a standard normal distribution, ensuring *β* = 0, no effect of the mediating trait on the target trait.

### Polygenic null trait simulation

In this scenario, we assumed SNPs have polygenic effects (*δ*_*k*_) on the target trait (*Y*) while maintaining *β* = 0. The target trait is simulated as $Y=\epsilon_{\text{twas}}=\sum_{k}X_{k}\cdot \delta_{k}+\epsilon$, where *X*_*k*_ represents the *k*th column of the *N* × *M* genotype matrix *X*, *δ*_*k*_ represents the direct SNP effects on the target trait sampled from a normal distribution, and *ϵ* is a normally distributed error term independent of *δ*_*k*_ and *X*.

To achieve the specified heritability, we divided the polygenic component *X*·*δ* by its standard deviation and multiplied it by $\sqrt{h^{2}}$. Similarly, we divided the error term *ϵ* by its standard deviation and multiplied by $\sqrt{1-h^{2}}$. The resulting target trait (*Y*) has variance 1, by construction. Our downstream results are not changed by this normalization, since we mainly focus on the *Z* score, which is independent of the scale of the target trait.

### Associations and test statistics

In both scenarios—polygenic and non-polygenic null—we carried out 1,000 simulation replicates. For every replicate, we generated scaled traits *T* and *Y* and computed the *Z*^2^ test statistic based on the correlation between *T* and *Y*. The correlation was obtained efficiently via matrix multiplication.^21^

To assess calibration of the *Z*^2^ values, we compared their empirical distribution to the theoretical $\chi_{1}^{2}$ (chi-squared with one degree of freedom) using quantile-quantile (QQ) plots. This comparison reveals whether type I error is properly controlled under the null hypothesis.

We also examined the calibration of the sample mean of the *Z*^2^ statistics across the 1,000 replicates. Specifically, we calculated the empirical mean of the *Z*^2^ values and compared it against the distribution of theoretical sample means $\chi_{1}^{2}$. We generated a figure that shows a histogram of the expected sample means with a vertical line indicating the observed mean; the overlay provides a clear visual benchmark for any systematic deviation from the null expectation.

### Sampling genotype data from the UK Biobank

We processed imputed UK Biobank Binary Genotype (BGEN) files from all chromosomes, extracting specific variants and samples for downstream genetic analysis. We obtained the UK Biobank sample list and excluded individuals who had withdrawn consent and all related individuals, retaining 377,620 unrelated individuals. From these, we created different subset sample sizes (1,000, 4,000, 5,000, 8,000, 10,000, 12,000, 16,000, 18,000, 20,000, and 100,000) through random sampling. We selected non-ambiguous SNPs from HapMap3 with MAF > 0.01 among European-descent individuals.^22^ A total of 1,108,189 variants passed selection criteria. We excluded individuals with genotype missingness >1%.

### Simulation of null traits in the UK Biobank

We simulated polygenic null target traits following Equation 1 with *β* = 0 and *ϵ*_twas_ = ∑_*k*_*X*_*k*_·*δ*_*k*_ + *ϵ*. We varied sample sizes and heritability ($h_{\delta }^{2}$) across simulations. The vector *δ* has length *M* = 1,108,189 (matching the HapMap3 SNPs), with effect sizes drawn from a normal distribution. We assume HapMap3 SNPs are in close LD with causal variants and serve as good proxies for target trait effects.

Using the same strategy as the minimal simulation above, we normalized the target trait (*Y*). We divided the polygenic component ∑_*k*_*X*_*k*_·*δ*_*k*_ by its standard deviation and multiplied by $\sqrt{h^{2}}$. We also divided the error term (*ϵ*) by its standard deviation and multiplied by $\sqrt{1-h^{2}}$ such that the resulting target trait (*Y*) has variance equal to 1, by construction. Once again, our downstream results are based on *Z* scores, which are unaffected by this normalization.

### Simulation of alternative traits for power analysis

We simulated traits under an alternative model that incorporates polygenic effects, gene-level effects, and an error term. The simulated trait is defined as *Y* = ∑_*k*_*X*_*k*_⋅*δ*_*k*_+∑_*g*_*T*_*g*_⋅*β*_*g*_+*ϵ*, where *X*_*k*_ represents the genotype matrix, *δ*_*k*_ represents direct SNP effects, *T*_*g*_ is the predicted gene-expression matrix, *β*_*g*_ is the effect size for genes where *g* indexes the genes, and *ϵ* is a normally distributed error term.

We simulated the polygenic component ∑_*k*_*X*_*k*_·*δ*_*k*_ as described for the null trait in the minimal example above with the proportion of variance explained by the polygenic component set to $h_{\delta }^{2}=0.5$.

To predict expression, we used whole-blood prediction model weight and genotype of the selected 100,000 UK Biobank individuals. We uniformly sampled a varying number of causal genes (3, 10, or 100) from the complete set of predicted genes. We assigned the selected causal genes a fixed nonzero effect size (*β*_*g*_ ≠ 0) while all non-causal genes were assigned effect sizes of zero (*β*_*g*_ = 0). Gene-level contributions were computed as ∑_*g*_*T*_*g*_·*β*_*g*_, standardized, and scaled using $h_{g}^{2}=0.05$ to ensure we achieved the desired variance explained by gene expression for the trait (Y). We used the approach described previously: we divided the gene-level effect ∑_*g*_*T*_*g*_·*β*_*g*_ by its standard deviation and multiplied by $\sqrt{h_{g}^{2}}$.

The residual error term (*ϵ*) was also modeled as previously described. We divided the error term (*ϵ*) by its standard deviation and multiplied by $\sqrt{1-h_{\delta }^{2}-h_{g}^{2}}$ such that the resulting trait has a total variance equal to 1.

We performed association tests between predicted gene-expression levels and the simulated phenotype, yielding gene–trait *Z* scores and corresponding *p* values.

### Sensitivity analysis to varying proportion of SNPs with nonzero effects

We conducted a simulation study to evaluate the sensitivity of our method to the proportion of selected HapMap3 SNPs from UK Biobank with nonzero direct effect (*δ*_*k*_ ≠ 0) on the target trait.

The polygenic null traits are simulated following the same procedure as described above with the modified vector of direct effects *δ*_*k*_. To achieve the desired proportion on nonzero effect sizes, we multiply a random subset of *δ*_*k*_ by 0. The vector *δ* maintains the same length.

We simulated phenotypes for 100,000 individuals from the UK Biobank with a polygenic heritability of $h_{\delta }^{2}=0.8$ and varied the proportion of SNPs with nonzero effects (0.3, 0.5, and 0.9). We chose a high value for $h_{\delta }^{2}$ to test the robustness of our method in extreme cases. We performed association tests between predicted gene expression and the simulated phenotype to obtain *Z* scores and *p* values. To correct for potential inflation, we applied both the BACON^12^ and our variance-control methods to the association results. Finally, we compared the results with the QQ plot of the corrected and uncorrected *p* values against the expected null (uniformly distributed between 0 and 1).

### Prediction of mediating traits

#### Predicting gene expression

We used gene-expression prediction weights from PredictDB (https://predictdb.org/),^1^ a widely used repository for predicting gene expression and TWAS. From PredictDB, we downloaded gene-expression model weights for 49 tissues and used PrediXcan software to impute gene expression from UK Biobank genotypes. For comparison, we downloaded whole-blood gene-expression weights from Fusion (http://gusevlab.org/projects/fusion/),^2^ another commonly used TWAS method, converted them to PredictDB format, and used them for gene-expression imputation. Although we did not use it, OmicsPred (www.omicspred.org/)^23^ is another popular repository that contains prediction weights for different molecular traits that could be used for this purpose.

#### Predicting phenotypes from MRI

We downloaded phenotype prediction models trained on UK Biobank MRI-derived data from the PredictDB repository and used BrainXcan software to impute predicted brain features.^24^

#### Predicting metabolite levels

We used in-house-trained metabolite-prediction models from the Metabolic Syndrome in Men (METSIM) dataset^25^ to impute metabolite levels in UK Biobank genotypes.

### Characterizing TWAS inflation as a function of sample size and heritability and estimation of the inflation slope Φ

To investigate the impact of TWAS sample size and heritability of the target trait on inflation, we tested the associations between polygenic null traits and predicted mediators for a range of sample sizes and heritabilities. We used the UK Biobank genotype data to predict gene expression, metabolites, and brain features following the procedure described above.

For a given sample size and heritability pair, we simulated 1,000 polygenic null target traits and tested the association between *Y* and the mediating trait. We collected the square of the *Z* scores (*Z*^2^) for each association and computed the average across the 1,000 simulations as a proxy for the expected value of the *Z*^2^.

We repeated this procedure for all combinations of sample sizes (*N* = 2,000, 6,000, 10,000, 14,000, 16,000, 18,000, 20,000) and heritability ($h_{\delta }^{2}$ = 0, 0.25, 0.5, 0.75, 1).

We show the result for one representative molecular trait from each category (gene = *KANSL*, metabolite = 3-ethylphenylsulfate, brain feature = IDP-25676) in Figure 3, where the linear relationship between the inflation (*EZ*^2^) and sample size and heritability is apparent.

We estimated the slope of the inflation growth as a function of the sample size and heritability by regressing *EZ*^2^ against $Nh_{\delta }^{2}$. We performed one regression for each mediator, including 281,488 gene-tissue pairs from the GTEx, 580 metabolites, and 471 brain features. Acknowledging the uncertainty in the slope estimation, we adjusted the estimated slope Φ by adding one standard error to conservatively account for potential estimation error.

### Inflation correction method

Given the uncorrected TWAS *Z* score and slope parameter for each gene (or other mediating trait) estimated in the previous section, we perform the correction by dividing the *Z* score by

$$Z_{corr}=\frac{Z_{twas}}{\sqrt{Var\left(Z_{twas}\right)}}=\frac{Z_{twas}}{\sqrt{\left(1+\Phi_{gene}*N*h_{\delta }^{2}\right)}}$$

The sample size (*N*) should be provided as part of the GWAS results, and the heritability is estimated from the GWAS results. This method has been added to the PrediXcan standard software.

### TWAS of 110 GWAS traits using harmonized summary statistics

To investigate inflation in real-world TWAS results, we analyzed GWAS summary statistics that were harmonized previously.^26^ Briefly, variants were mapped to the GRCh38 reference genome; for multiallelic sites, the allele with the highest MAF was selected. The processed GWAS was imputed with *Z* scores using the best linear unbiased prediction (BLUP) approach for variants reported in GTEx but not available in the summary statistics.

We estimated the heritability of each GWAS trait using LDSC,^18^ with LD scores computed from European-ancestry samples in the 1000 Genomes Project (EUR) and using default parameters. We used the GWAS sample size as reported in the study.

We performed TWAS using S-PrediXcan,^27^ which integrates GWAS summary statistics with gene-expression prediction models trained in whole blood from GTEx. After obtaining the association *Z* scores, we applied our variance-control adjustment by dividing each *Z* score by $\sqrt{1+Nh_{\delta }^{2}\Phi }$, where *N* is the GWAS sample size, $h_{\delta }^{2}$ is the trait heritability, and Φ is the inflation factor estimated for each gene. The adjusted *Z* scores were then used to compute corrected *p* values.

## Results

### No inflation for non-polygenic null traits

To establish a baseline, we first tested TWAS calibration under ideal conditions using simulated data. We simulated unrelated target traits (*Y*) and mediating traits (*T*), where the target trait has no polygenic component (termed non-polygenic null).

We generated non-polygenic null traits for 1,000 individuals by sampling from a normal distribution. For mediating traits, we simulated 999 independent SNPs with genetic effects (*γ*_*k*_) drawn from a standard normal distribution, then computed *T* as a weighted average of SNP dosages ($\sum_{k}X_{k}\gamma_{k}$). We repeated this simulation 1,000 times, regressing *Y* on *T* and computing *Z* scores for each iteration. To ensure robustness, we also performed simulations using t-distributed traits and effects (Figures S1A–S1C).

Under proper calibration, *Z* scores should follow a standard normal distribution with sample variance approaching 1; values significantly exceeding 1 indicate test inflation.

**Figure 1:**
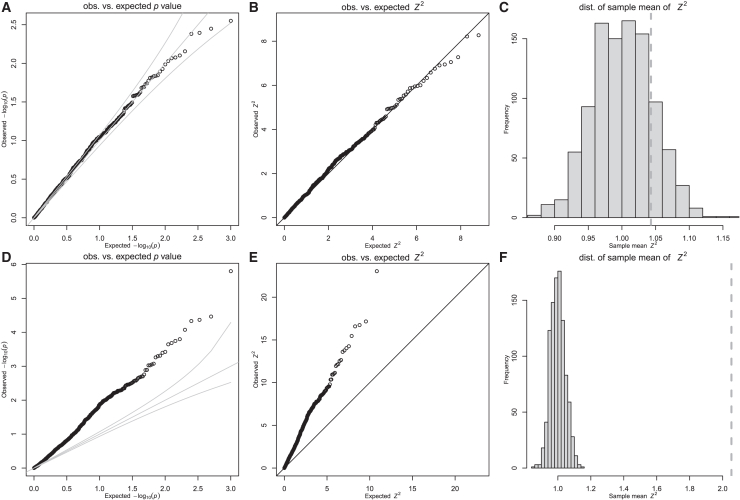
Inflation of simulated TWASs under minimal conditions Simulated TWAS results under the null hypothesis using 999 SNPs with MAF = 0.4 across *N* = 1,000 individuals, with effect sizes and errors sampled from normal distributions. (A–C) Non-polygenic null trait simulations (*Y* = *ϵ*);(D–F) polygenic null trait simulations ($Y=\sum_{k}X_{k}\cdot \delta_{k}+\epsilon$): (A and D) QQ plot of observed vs. expected *p* values, (B and E) QQ plot of observed vs. expected *Z*^2^, and (C and F) distribution of expected sample averages of squared standard normal random variables. Dotted vertical line indicates average *Z*^2^ for 1,000 simulations. We provide a shinyapp at https://imlab.shinyapps.io/twas-inflation/, which can be used to replicate these results and perform visualization using different parameters.

As expected, association statistics between the mediator (*T*) and non-polygenic null traits (*Y*) are well calibrated, following the theoretical null distributions (uniform for *p* values and $\chi_{1}^{2}$ for test statistics) (Figures 1A and 1B). The average *Z*^2^ across the 1,000 iterations falls within the expected distribution of sample means (*n* = 1,000) drawn from a $\chi_{1}^{2}$ distribution (Figure 1C).

### No inflation with error in prediction independent of trait

In practice, we do not know the “true” weights for the genetic component of the mediator (*T*), meaning in our simulations we use a set of weights (${\tilde{\gamma }}_{k}$) that differ from the true values (*γ*_*k*_). This includes cases where *γ*_*k*_ is zero but ${\tilde{\gamma }}_{k}$ is not and vice versa. Since prediction weights are typically trained in studies independent of the GWAS, it is reasonable to assume that the prediction error will be independent of the error term in the target trait *Y* (i.e., *ϵ*_twas_ under the null). Under this assumption, the error-in-variable literature states that the association test using a noisy explanatory variable remains valid, meaning there is no inflation of type I error.^15^

This result is fairly intuitive: under the null, a condition where the mediator (*T*) is unrelated to the target trait (*Y*), adding error to *T* is unlikely to strengthen the association, provided the prediction error is independent of the target trait. If, however, prediction errors were systematically associated with target traits and this effect was large, TWAS results would be invalid, and the field would need to halt the use of the approach until a solution is developed. That said, most researchers would likely view this as an extreme measure and would agree that assuming independence between prediction error and the target trait under the null is reasonable.

### Polygenic null target trait causes inflated type I error

To evaluate the impact of direct SNP effects on the target trait on type I error calibration, we simulated polygenic null traits ($Y=\epsilon_{\text{twas}}=\sum_{k}X_{k}\cdot \delta_{k}+\epsilon$) and mediating traits ($T=\sum_{k}X_{k}\cdot \gamma_{k}$). As described in the methods section, *δ*_*k*_ are sampled from a normal distribution with mean of 0 and variance of $\sigma_{\delta }^{2}$, *X*_*k*_ are simulated genotype dosages, and *ϵ* are independent normally distributed random variables. We also performed the simulation using t-distributions to check robustness to deviations from normality (see Figures S1D–S1F).

If this polygenic component is independent of the mediator (*T*), the usual regression assumption holds and we would not expect inflated type I error, i.e., we would expect that the variance of the *Z* score statistic has variance of 1. However, contrary to our intuition, the sample variance of the *Z* score statistic was much larger than its expected distribution, as shown in Figures 1D–1F. This inflation is observed for a range of parameters as can be visualized using this shinyapp (https://imlab.shinyapps.io/twas-inflation/) or using the code in Code S1.

### Polygenic null target traits yield inflation using real-world predicted expression data

To test whether TWAS inflation occurs in a more realistic scenario, we predicted the expression of 7,131 genes in whole blood for individuals in the UK Biobank and tested the association with polygenic null traits.

We chose to use simulated polygenic null traits rather than actual phenotype data because none of the phenotypes available in the UK Biobank can be considered null; they all yield multiple genome-wide significant loci, suggesting that some genes may have an effect on the phenotype, either directly or through confounders.

As described in the methods section, for non-polygenic null traits, we sampled values from a normal distribution. For polygenic null traits, we used UK Biobank genotype data and constructed each trait as a linear combination of genotype dosages with randomly generated weights (*δ*_*k*_) plus random noise (*ϵ*), all from the normal distribution. We show results for the *AMT* gene as a randomly selected representative example. Other genes and tissues behave similarly to *AMT* in both non-polygenic and polygenic null trait simulations.

**Figure 2:**
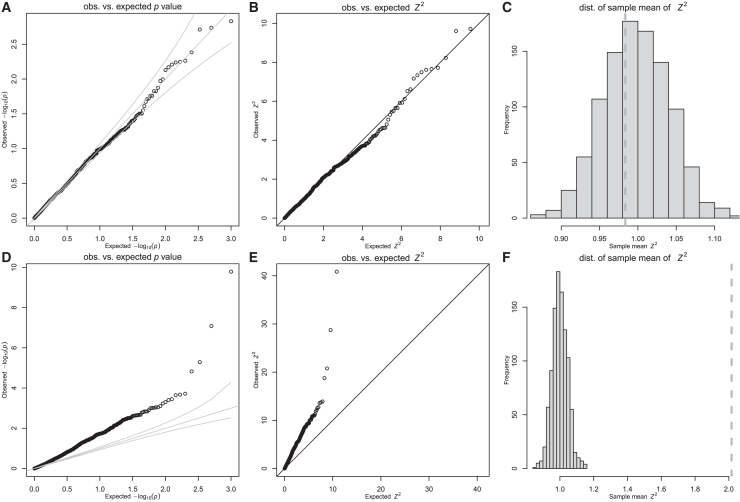
Inflation in TWAS with real predicted expression in the UK Biobank Simulated TWAS results using real-world predicted expression of a representative gene (*AMT*) and simulated null target traits in whole blood using genotype data of 10,000 randomly sampled unrelated white British individuals from the UK Biobank. Prediction weights for *AMT* were downloaded from PredictDB.org. (A–C) Non-polygenic trait simulations and (D–F) polygenic trait simulation: (A and D) QQ plot of observed vs. expected *p* values, (B and E) QQ plot of observed vs. expected *Z*^2^, and (C and F) distribution of expected sample averages of squared standard normal random variables. Dotted vertical line indicates average *Z*^2^ for 1,000 simulations.

For non-polygenic null traits, TWAS *p* values, squared *Z* scores, and sample variances all followed expected distributions, showing no type I error inflation (Figures 2A–2C). Since these predicted expressions are all noisy versions of the true mediator, these results provide another confirmation that noisy gene-expression predictors do not cause an inflation in type I error for non-polygenic null traits.

For polygenic null traits, however, we observed significant deviations from expected distributions in *p* values, *Z* scores, and sample variance of the *Z* scores (Figures 2D–2F). Moreover, similar inflation was observed using the Fusion software and prediction weights from the Fusion/TWAS website (Figure S2), indicating that this issue is intrinsic to the TWAS approach rather than specific to any particular implementation. We also verified that the substance of our findings did not change when using different tissue expression or relaxing the normality assumption for effect sizes and error terms (see Figure S3).

### Inflation grows linearly with the trait heritability and the GWAS sample size

**Figure 3:**
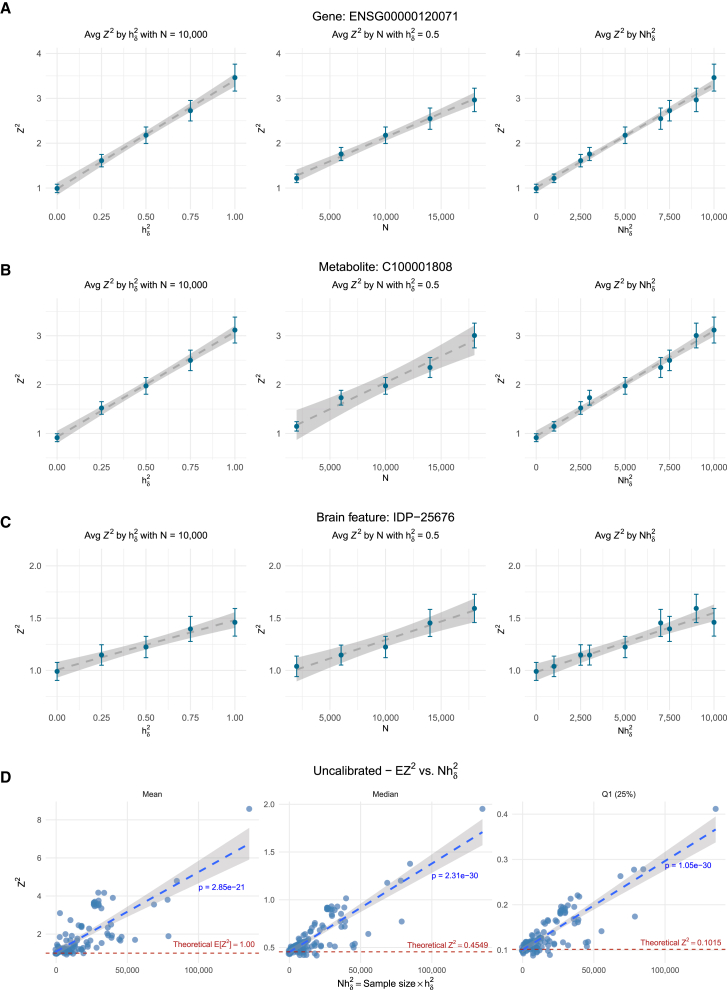
Inflation grows linearly with the GWAS sample size and heritability of the target trait (A–C) The average *Z*^2^ is plotted against heritability ($h_{\delta }^{2}$), sample size (*N*), and the product of heritability and sample size ($Nh_{\delta }^{2}$). Each dot represents the average association of predicted expression of (A) *KANSL1* gene, (B) 3-ethylphenylsulfate (*C100001808*) metabolite, and (C) MRI of *IDP-25676* with UK Biobank genotype data and 1,000 polygenic null traits in the same individuals at the specified sample sizes and heritability values. (D) Inflation in real TWAS of 110 GWAS traits showing the mean, median, and first quantile of *Z*^2^ across genes for each trait against $Nh_{\delta }^{2}$. In contrast to (A–C), each dot in (D) represents a GWAS trait. The error bars in (A–C) show 1.96× the standard errors of the sample averages. Dashed lines correspond to estimated linear regression lines based on the *Z*^2^, and the gray band represents the confidence interval of the regression lines.

We examined the relationship between the sample variance of the *Z* score ($EZ_{\text{twas}}^{2}$) and both sample size and target trait heritability using 1,000 simulated polygenic null traits in the UK Biobank for various combinations of sample size and heritability. Figure 3A shows that the sample variance of the *Z* score increases linearly with both factors. When the trait has no polygenic component ($h_{\delta }^{2}=0$), the sample variance is approximately 1, as expected. However, the rate of inflation varied across genes, suggesting a straightforward formula for predicting the variance of the *Z* score:

(Equation 2) $$E\left[Z_{\text{twas}}^{2}\right]\approx 1+Nh_{\delta }^{2}\Phi \left(\text{gene}\right)\text{,}$$

where Φ is the slope of inflation as a function of $Nh_{\delta }^{2}$.

In fact, our theoretical derivation—based on commonly used assumptions in statistical genetics (see supplementary note)—demonstrates that $\text{var}\left(Z_{\text{twas}}\right)=E\left[Z_{\text{twas}}^{2}\right]$ aligns with the formula in Equation 2 with an “inflation slope parameter” given by the following expression:

(Equation 3) $$\Phi \left(\text{gene}\right)=\frac{1}{M}\frac{{\tilde{\gamma }}^{\prime }\cdot \Sigma^{2}\cdot \tilde{\gamma }}{{\tilde{\gamma }}^{\prime }\cdot \Sigma \cdot \tilde{\gamma }}\text{,}$$

where Σ is the genome-wide LD matrix, *M* is the effective number of causal SNPs for the target trait, and $\tilde{\gamma }$ is the prediction weights vector downloadable from various publicly available databases (e.g., Fusion, predictdb.org, omicspred.org). $\tilde{\gamma }$ is considered to be genome-wide but entries without weights are set to 0 to match the LD matrix dimension.

**Figure 4:**
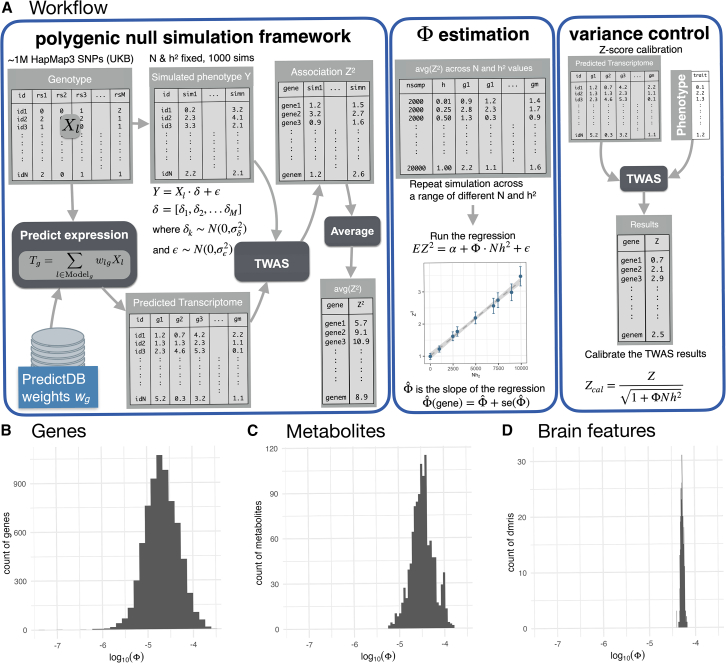
Inflation slope estimation and variance-control correction workflow (A) The workflow has three sections: polygenic null simulation framework, where we simulate the polygenic null trait and run TWAS to obtain association statistics; Φ estimation, where we perform multiple simulations varying the combination of N and $h_{\delta }^{2}$ followed by running linear regression for each gene to obtain the $\hat{\Phi }$; and variance control, where we calibrated TWAS results using our method. (B–D) Distributions of estimated inflation factors Φ for (B) gene expression, (C) metabolites, and (D) brain features (diffusion MRI) are shown in the *log*_10_ scale. The factor Φ for each mediator is estimated using the average *Z*^2^ statistics of the association between genetically predicted mediator and 1,000 simulated target traits for each combination of heritability of target trait $h_{\delta }^{2}$ and sample size *N*. The slope of the regression of E*Z*^2^ on $Nh_{\delta }^{2}$ is used to estimate Φ. Most values (78% of genes, 94% of metabolites, 100% of brain features) are on the order of 10^−5^.

In practice, this formula cannot be used to estimate inflation because of the challenges associated with accurately estimating the effective number of causal variants (*M*), the true LD matrix, and validating key assumptions. Hence, we opted for an empirical approach as described below and illustrated in Figure 4A.

### Inflation in other xWASs has similar properties

The mathematical modeling of the genetic component of gene expression is identical to the modeling of metabolite levels and brain features (weighted average of SNP effects), implying inflation in xWAS results would also have linear dependence on sample size and heritability in Equation 2. However, metabolites and brain features have different genetic architectures than gene expression. While the most predictive SNPs for gene expression are typically located near the gene itself, metabolites and brain features are predicted using genome-wide variants, and their prediction models generally require a larger number of nonzero weights compared to those for gene expression. These differences motivated us to investigate how increased predictor dispersion and polygenicity of the mediating trait affect the magnitude of the inflation.

We performed the association between metabolite levels/brain features and polygenic null traits following the procedure outlined for gene expression in the previous section. Briefly, using the same polygenic null traits simulated in the gene-expression section, we correlated 580 genetically predicted metabolite levels and 471 brain features with the polygenic null target trait. We found that, for all features, $EZ_{\text{twas}}^{2}$ is greater than 1, indicating inflation, and that it increases linearly with the GWAS sample size and heritability. In Figures 3B and 3C, we show one representative example metabolite and brain feature, but all others followed a similar pattern, with an inflation slope (Φ) specific to each metabolite and brain feature.

### Real TWAS also shows inflation growing linearly with sample size and heritability

We investigated TWAS inflation in real data, using summary statistics from 110 previously harmonized GWASs,^26^ covering a broad range of complex-trait classes—including cardiometabolic, anthropometric, psychiatric, autoimmune, respiratory, and hematologic traits (see Table S1).

We performed TWAS with whole-blood predicted gene expression trained on GTEx, using the summary-based PrediXcan software.^27^ For each trait, we estimated $E\left[Z_{\text{twas}}^{2}\right]$ by averaging the squared *Z* statistics across genes and plotted these averages against the product of sample size (*N*) and trait heritability ($h_{\delta }^{2}$) (Figure 3D). Consistent with our simulations and theoretical expectations, $E\left[Z_{\text{twas}}^{2}\right]$ increased linearly with $Nh_{\delta }^{2}$. A linear regression of the mean *Z*^2^ on $Nh_{\delta }^{2}$ gives

$$E\left[Z_{\text{twas}}^{2}\right]\approx 1.1+N\;h^{2}\;4.2\times 10^{-5}\text{,}$$

with a Wald-test *p* value of 10^−21^ and an *R*^2^ of 0.56, indicating that $Nh_{\delta }^{2}$ explains 56% of the variation in $E\left[Z_{\text{twas}}^{2}\right]$. The intercept (1.1) is slightly above 1, likely reflecting contributions from truly causal genes. The slope (4.2 × 10^−5^) quantifies the magnitude of inflation per unit increase in $Nh_{\delta }^{2}$, indicating that inflation will start being noticeable at sample sizes above 10^5^.

To ensure that this linear trend is not driven solely by causal genes, we examined the median and lower quartile of the *Z*^2^ distribution for each GWAS trait (Figure 3D). These lower-quartile statistics are dominated by non-causal genes, yet they also follow the same linear pattern (see Table S2 for additional quartiles of both uncalibrated and calibrated *Z*^2^ values). This indicates that the observed inflation is a general property of TWAS statistics rather than an artifact attributable to a subset of causal genes.

### Estimating the inflation factor

To estimate the slope parameter Φ—a property of each gene or mediator—we followed the workflow illustrated in Figure 4A. A step-by-step guideline for estimating Φ is publicly available on GitHub wiki (https://github.com/hakyimlab/twas-inflation/wiki/Estimating-phi-for-variance-control-method). For every predicted gene or mediating trait, we simulated 1,000 polygenic null phenotypes (*Y*) for a grid of sample sizes and heritability values (methods), conducted association tests on each simulated phenotype, and averaged the corresponding *Z*^2^ values across the 1,000 replicates for every combination of sample size and heritability. We then estimated Φ by regressing the mean *Z*^2^ on $Nh_{\delta }^{2}$. To incorporate estimation uncertainty, we added one standard error to the fitted slope; this conservative choice yields a more stringent correction.

We analyzed three collections of predictors: protein-coding gene-expression models in 49 GTEx tissues,^26^ 580 metabolite-prediction models trained on METSIM data,^25^ and 471 brain features from the UK Biobank.^24^

The estimated Φ values differed across genes, metabolites, and brain features. As shown in Figures 4B–4D, gene expression exhibited the broadest range of Φ (0–2 × 10^−4^, median 2 × 10^−5^), followed by metabolites (0–1.5 × 10^−4^, median 3.3 × 10^−5^) and brain features (3.9 × 10^−5^ – 6.5 × 10^−5^, median 5.3 × 10^−5^).

The majority of mediating traits—78% of genes, 94% of metabolites, and 100% of brain features—had Φ values on the order of 10^−5^, comparable to the slope obtained from the 110 real TWASs (Φ = 4.2 × 10^−5^). This concordance indicates that the inflation patterns observed in simulations are also present in real data.

### Variance-control strategy to correct for inflation

TWAS/xWAS *p* values are usually computed by assuming that, under the null hypothesis, the *Z* scores follow a standard normal distribution, *N*(0,1). As shown above, however, the variance of the *Z* scores exceeds 1 for polygenic target traits; a broader distribution with more extreme *Z* score values yields *p* values lower than expected under the null, inflating significance. This inflation should be corrected to obtain reliable TWAS/xWAS results.

Our correction consists of scaling each *Z* score by the square root of its expected variance: $Z_{\text{corr}}=\frac{Z_{\text{twas}}}{\sqrt{E\left[Z_{\text{twas}}^{2}\right]}}=\frac{Z_{\text{twas}}}{\sqrt{\text{Var}\left(Z_{\text{twas}}\right)}}\text{.}$ The variance term (Equation 2) depends only on three quantities: the GWAS sample size *N*, the heritability of the target trait $h_{\delta }^{2}$, and the inflation slope Φ. The Φ for each mediating trait is estimated using the procedure described in the previous section.

**Figure 5:**
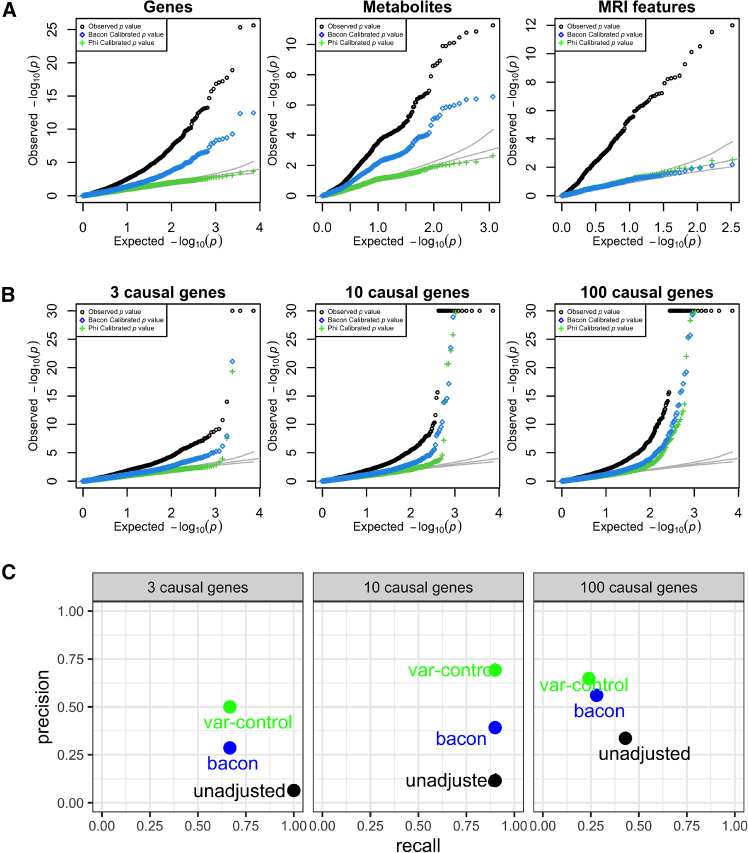
Our variance-control approach corrects inflation of xWAS in UK Biobank (A) QQ plot of the uncorrected (black), BACON-corrected (blue), and variance-controlled (green) −*log*_10_*p* values of the association between 7,131 genes, 580 metabolites, and 471 brain features and a polygenic null target trait in 100,000 UK Biobank individuals. The expected distribution is given by the identity line. This figure shows the results based on PredictDB models. Figure S4 shows similar inflation and correction effectiveness with Fusion models. (B) QQ plot of the association between predicted whole-blood expression and a polygenic trait with a specified number of causal genes (3, 10, and 100, left to right) in 100,000 UK Biobank individuals and the heritability of the polygenic component $h_{\delta }^{2}$ fixed at 0.5. (C) Precision and recall metrics calculated at the Bonferroni significance threshold (*p* = 0.05/7,131 = 7.0 × 10^−6^) using polygenic target traits with the specified number of causal genes (3, 10, and 100, left to right).

We evaluated our variance-control approach against BACON, an existing Bayesian-based method for controlling xWAS inflation. We tested the associations between 7,131 genes, 580 metabolites, and 471 brain features and a polygenic null target trait in 100,000 UK Biobank individuals (Figure 5A). In these types of traits, uncorrected *p* values show substantial inflation. While both variance control and BACON reduce inflation, only the variance-control method provides well-calibrated *p* values, closely aligning with the expected distribution.

### Variance control yields higher precision under the alternative hypothesis

To assess how variance control and the BACON correction affect precision (the probability that a gene is truly causal given that it is declared significant) and recall (power or the probability that a causal gene reaches significance), we added a small number of causal genes to our simulations.

We used the alternative traits simulation framework with varying numbers of causal genes (3, 10, or 100). We generated phenotype (*Y*) from 100,000 UK Biobank individuals using heritability $h_{\delta }^{2}=0.5$ for the polygenic component. In addition to the polygenic background, we added the contribution of the causal genes, $Y=T_{1}\beta_{1}+\cdots +T_{g}\beta_{g}+\sum_{k}X_{k}\cdot \delta_{k}+\epsilon \text{,}$ where *g* indexes the genes and *k* indexes the genetic variants. We defined a discovery set by applying a Bonferroni-corrected significance threshold (*p* = 0.05/7131 = 7.0 × 10^−6^). Genes with *p* values below this threshold were declared discoveries.

We performed TWAS on the simulated phenotype using predicted whole-blood expression from the same 100,000 individuals. The uncorrected, BACON-adjusted, and variance-controlled *p* value distributions for the three causal-gene scenarios are shown in Figure 5B. For the discovery sets, variance control achieved higher precision than both the uncorrected and BACON methods, without sacrificing recall (power) (Figure 5C). Recall for the variance-control approach was only slightly reduced relative to BACON for the simulation with the largest number of causal genes (24% versus 28%, respectively; 100 causal genes). These results indicate that variance control improves the reliability of TWAS/xWAS discoveries while preserving detection power.

### Robustness of Φ estimation

We verified that the estimated slopes were not influenced by the proportion of variants with nonzero effects (*δ*_*k*_ ≠ 0) in simulating the polygenic null trait by comparing estimates across varying proportions of such variants. We found that the slope estimations remained relatively stable as the proportion of nonzero SNPs varied (30%, 50%, and 80% of total SNPs; see Figure S6), although the estimated inflation slope for 30% nonzero SNPs is slightly smaller than that for 100% nonzero SNPs. However, since our chosen approach uses 100% of HapMap3 SNPs with nonzero effects, the slight overestimation of the inflation slope will lead to a more conservative inflation correction.

We also verified that the calibration of TWAS results is robust to varying proportions of SNPs with nonzero effects size. In contrast, the BACON method did not fully correct the inflation, as shown in Figure S7.

### Inflation correction in real TWAS applications using GWAS summary statistics

To evaluate how the corrections perform on real data, we applied both variance control and the BACON adjustment to the TWAS results for the 110 traits examined in the linearity-inflation analysis.

**Figure 6:**
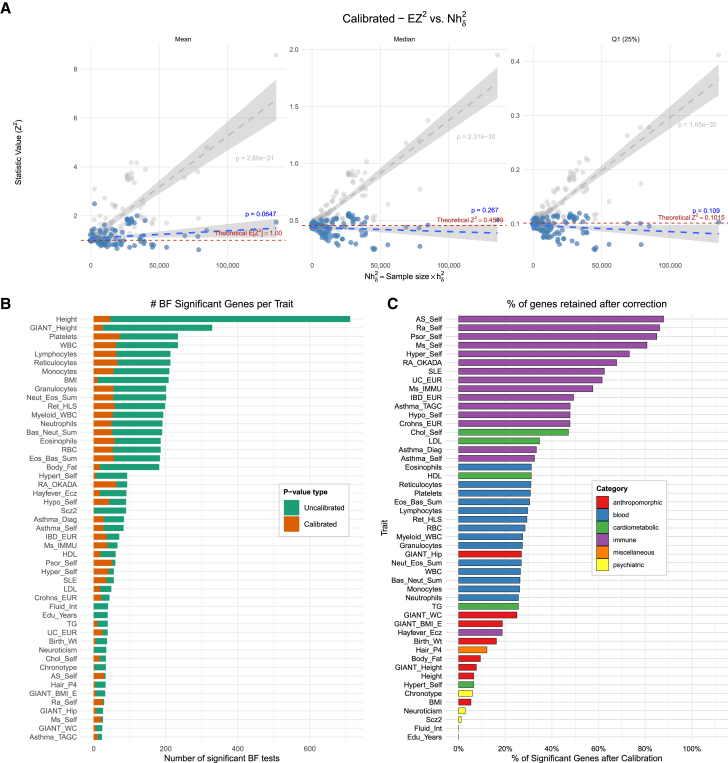
TWAS of real GWAS (A) Mean, median, and first quartile of the adjusted *Z*^2^ across genes for each trait plotted against $Nh_{\delta }^{2}$. The variance control removes the linear dependency of *Z*^2^ on $Nh_{\delta }^{2}$ with regression *p* values of 0.06, 0.26, and 0.10 for mean, median, and the first quantile of *Z*^2^, respectively. (B) Traits ranked according to the total number of Bonferroni (BF) significant genes (*p* = 7.0 × 10^−6^) before correction (green). Orange indicates the number genes that remain Bonferroni significant after correction with our variance-control method. (C) Proportion of uncalibrated Bonferroni-significant genes that remain Bonferroni significant after correction with our variance-control method, color coded by trait type. In (B) and (C), we only show traits that had 20 or more Bonferroni-significant genes.

After correction, the number of Bonferroni-significant genes changes markedly: the median number of significant genes for the uncorrected results is 12 (interquartile range [IQR] 2–61). BACON reduces this to a median of 9 (IQR 2–44), whereas variance control yields a median of 4 (IQR 0–21). Furthermore, the linear relationship between $Nh_{\delta }^{2}$ and the mean *Z*^2^ statistic disappears after correction (Figure 6A). The slopes from the regressions of *Z*^2^ on $Nh_{\delta }^{2}$ are 3.0 × 10^−6^ (*p* = 0.06) when using the mean *Z*^2^, −3.7 × 10^−7^ (*p* = 0.26) for the median *Z*^2^, and −1.1 × 10^−7^ (*p* = 0.11) for the first-quartile *Z*^2^.

Figure 6B shows the total number of Bonferroni-significant genes for each trait, with the total number reflecting the sample size of the GWAS study and the genetic architecture of the trait.

After calibration, immune-related traits (which typically involve a modest number of genes with relatively large effects) were only mildly affected and retained a larger portion of their significant genes. In contrast, highly polygenic traits, where the majority of genes have small effects^17^^,^^28^ (e.g., psychiatric disorders), experienced larger reductions in the count of significant genes after correction (Figure 6C).

### Theoretical derivation including nonzero mediating effect

We extended Equation 2 to evaluate how a nonzero mediator-target effect (*β* ≠ 0) influences $E\left[Z_{\text{twas}}^{2}\right]$. We also expressed the prediction weights as $\tilde{\gamma }=\gamma +\epsilon \text{,}$ where *γ* is the true weight vector and *ϵ* is an independent error term that is uncorrelated with both the true expression and the target trait. Under these assumptions (derivation in the supplementary note) the $E\left[Z_{\text{twas}}^{2}\right]$ can be written as

(Equation 4) $$E\left[Z_{\text{twas}}^{2}\right]\approx 1+\frac{N\;h_{\delta }^{2}}{1-\tau^{2}h_{\text{gene}}^{2}}\;\Phi +\frac{N\;\tau^{2}h_{\text{gene}}^{2}}{1-\tau^{2}h_{\text{gene}}^{2}}\text{.}$$

In this expression, *N* is the GWAS sample size, *β* is the true effect of the mediator (*T*) on the target trait (*Y*), $h_{\delta }^{2}$ is the polygenic component of *Y*, and Φ is the inflation slope defined in Equation 3. The vector $\tilde{\gamma }$ contains the noisy prediction weights; $\tau^{2}=\text{var}\left(T\right)/\text{var}\left(\tilde{T}\right)$ quantifies the precision of the mediator prediction (with $\tilde{T}$ the noisy version of *T*); $\sigma_{Y}^{2}$ and $\sigma_{T}^{2}$ are the variances of *Y* and *T*, respectively. Finally, $h_{\text{gene}}^{2}=\beta^{2}\sigma_{T}^{2}/\sigma_{Y}^{2}$ is the proportion of variance in *Y* explained by the mediator.

A useful corollary of Equation 4 is that prediction precision *τ*^2^ appears only together with $h_{\text{gene}}^{2}$ (i.e., as the product $\tau^{2}h_{\text{gene}}^{2}$). The inflation slope Φ itself does not depend on *τ*^2^; it is determined solely by the noisy weights $\tilde{\gamma }$, the LD matrix, and the number of predictors (*M*).

Consequently, under the null hypothesis ($h_{\text{gene}}^{2}=0$), prediction precision has no impact on type I error (see Figure S5A). The formula also shows that error in the predictor does not generate inflation. However, reduced precision lowers power because a smaller *Z*^2^ variance makes it harder to reach significance when $h_{\text{gene}}^{2}\neq 0$ (Figure S5B).

### PrediXcan software updated with correction method

To make the implementation of the correction user-friendly and straightforward, we provide inflation slope Φ estimates for gene-expression predictors in 49 GTEx tissues,^26^ 580 metabolite predictors from the METSIM study,^25^ and 471 brain features.^24^ We have also integrated these parameters into the database of gene-expression predictors (accessible at https://predictdb.org). To facilitate the implementation of variance-control correction, we updated the S-PrediXcan software (available at https://github.com/hakyimlab/MetaXcan) to automatically apply the correction using the inflation slope parameter Φ, with the output containing both the raw and corrected association statistics. These enhancements simplify the correction process for end users, who only need to provide the GWAS sample size and the heritability of the target trait. GWAS sample sizes are usually available with the study, and the heritability can be easily estimated from summary statistics using LDSC or similar methods.^18^

## Discussion

We report the problem of inflation of type I error (false-positive rate) in TWAS and other xWAS methods when the target traits are highly polygenic, both in simulations and in realistic TWAS settings with mediators predicted from UK Biobank data. This inflation is substantial and systematic: in our analysis of 110 GWAS traits, a large portion of initially significant associations became non-significant after correction for highly polygenic traits. Given the pervasive polygenicity of most complex traits, correcting this effect is critical for ensuring the reliability of TWAS/xWAS results. We therefore provide a user-friendly variance-control approach to correct for this inflation.

We demonstrate that the inflation is not exclusive to any single implementation of TWAS but applies to the entire class of methods that correlate genetic predictors of any mediating trait (e.g., metabolites, brain features) with a complex, polygenic trait. Consequently, any method investigating polygenic traits—such as PrediXcan,^1^ Fusion,^2^ PWAS,^29^ UTMOST (Unified Test for Molecular Signatures),^30^ and many others^9^^,^^10^^,^^11^—will yield an inflated false-positive rate with current GWAS sample sizes if left uncorrected. Analyses that correlate polygenic risk scores of biomarkers or other traits with a highly polygenic target trait suffer from the same inflation problem, highlighting the breadth of this issue.

We note that transcriptome-wide Mendelian randomization (TWMR), as an approach closely related to TWAS, is also inflated by polygenicity. In particular, the inverse variance-weighted fixed-effect estimate is inflated similar to TWAS. The random-effect-based approach naturally models polygenicity by introducing random effects, but some residual inflation is still observed when the number of instruments is small. In such cases, the random effect is harder to estimate accurately and is likely underestimated. To account for polygenicity-induced inflation in TWMR, we recommend generating polygenic null for TWMR estimates.

Inflation occurs across mediating traits with a variety of genetic architectures, including gene expression, metabolite levels, and MRI-derived brain phenotypes, which range from highly sparse (gene expression) to highly polygenic (brain features). More sparse traits such as gene expression tend to exhibit a wide spread of inflation parameters, while the polygenic traits show a narrow spread. Overall, the median inflation parameter is higher in polygenic traits.

We propose an effective strategy to correct for the inflation by estimating an inflation slope parameter that is specific to the mediator and valid for a large class of target traits, provided the target trait can be well approximated by an infinitesimal model in which the contribution of any single SNP is modest. Further research may improve the calculation of the inflation factor when the polygenic architecture of the target trait is more complex.

We have updated the PrediXcan software and its database of gene-expression prediction models to facilitate implementation of our correction method for the broad user base.

We also provide corroboration of a known fact from the error-in-variables literature: prediction error for gene expression does not cause inflation in type I error as long as the prediction error is independent of the target trait. This assumption of independence is reasonable because the prediction models are trained on studies that are independent of the GWAS data used for association. If this assumption fails, TWAS and related methods should be abandoned until a solution is found; we believe most researchers would view this as an extreme measure and would agree that assuming independence between prediction error and the target trait under the null hypothesis is reasonable.

Some may interpret the observed inflation as resulting from horizontal pleiotropy (i.e., variants that contribute to the prediction of the mediating trait also influence the target trait through mechanisms unrelated to the mediator). In our context, this horizontal pleiotropy arises from the polygenicity of the target trait. If every variant has an effect on the target trait or is in LD with a causal variant, then pleiotropy becomes unavoidable. We therefore distinguish between polygenic pleiotropy and the more commonly addressed form of local pleiotropy. In the polygenic case, the effects of SNPs on the trait are downstream consequences of a complex network of cascading processes, not attributable to a single gene, protein, or other molecular trait; they represent the accumulation of many small effects. By contrast, in local pleiotropy—a focus of most current studies—a variant has a relatively large effect on a focal gene expression (or is in LD with an eQTL for that gene) and also affects another gene, protein, or mediating trait.^6^^,^^7^^,^^8^^,^^31^ The two forms of pleiotropy are primarily distinguished by effect size. In the present paper, we address inflation due to polygenic pleiotropy; once a signal above this background is identified, alternative methods can be applied to narrow down the causal mediator.

Our study has several limitations. First, we assumed an additive infinitesimal model for the target trait in both simulations and theoretical derivations. In practice, traits may deviate from this model. Although the estimation of the inflation factor Φ could be refined for different genetic architectures, we expect the infinitesimal-model approximation to provide a valuable first-order correction. We observed a slight overestimation of the inflation slope when a low proportion of SNPs have nonzero effect on the target trait; this can be corrected by simulating a null phenotype that better matches the true genetic architecture of the target trait. Second, since our approach corrects for the polygenic background, only effects that are much larger than the polygenic background can be detected. Third, our correction does not account for horizontal pleiotropy with effect sizes larger than those assumed in the polygenic background. Co-regulation of multiple genes by the same variants and LD contamination are also not addressed by our method. While other approaches exist to tackle these issues, they each rely on additional assumptions.^6^^,^^7^^,^^8^ Because each method has its own advantages and limitations, we believe that polygenicity-corrected results should be considered as part of a broader set of analyses when drawing reliable conclusions about the function of GWAS loci.

Finally, our theoretical derivations were based on a linear regression framework for the TWAS test statistic. In our framework, we assume that disease traits are continuous. However, for binary traits, GWASs are typically conducted using logistic regression. As a result, when applying our variance-control correction to TWAS/xWAS results that rely on such GWAS summary statistics, the underlying linear approximation may be imperfect. Nevertheless, linear regression provides a good approximation for logistic regression when the case-control ratio is balanced; therefore, we expect our results to be broadly applicable to balanced designs. For unbalanced designs, however, the method will need to be modified.

## Data and code availability

• The code used to perform our inflation analysis is available in GitHub: https://github.com/hakyimlab/twas-inflation.
• We provide a shinyapp at https://imlab.shinyapps.io/twas-inflation/, which can be used to replicate these results and perform visualization using different parameters.
• We provide updated prediction models with Phi at www.predictdb.org.

## Acknowledgments

This research has been conducted using the UK Biobank Resource under application number 89052. This research used resources of the Argonne Leadership Computing Facility, which is a DOE Office of Science User Facility supported under contract DE-AC02-06CH11357. This work was completed in part with resources provided by the University of Chicago’s Research Computing Center and Beagle3. We also acknowledge resources from the Center for Research Informatics, funded by the Biological Sciences Division at the 10.13039/100007234University of Chicago, with additional funding provided by the Institute for Translational Medicine, 10.13039/100016220CTSA grant number 2U54TR002389-06 from the 10.13039/100000002National Institutes of Health. We thank Sarah Sumner for help editing the paper. The following grants provided partial support to this project: R01AA029688, P30DK020595, and 3R01CA242929-04S1. UK Biobank genotype data were obtained under application number 89052. The updated MetaXcan software (v0.8.1) is available on GitHub and Zenodo.

## Declaration of interests

The authors declare no competing interests.

## Web resources

Fusion, http://gusevlab.org/projects/fusion/.
MetaXcan (v0.8.1) on GitHub, https://github.com/hakyimlab/MetaXcan/releases/tag/v0.8.1.
MetaXcan (v0.8.1) on Zenodo, https://doi.org/10.5281/zenodo.15722727.
PredictDB, http://predictdb.org.
UK Biobank, https://www.ukbiobank.ac.uk/n.

## Supplemental information

Document S1. Figures S1–S13 and supplemental notes

Table S1. Metadata of the 110 GWAS traits used to perform TWASs The table contains the GWAS name, source, sample size, and category of each trait.

Table S2. The uncalibrated and calibrated *Z*^2^ distributions for each trait The table contains the mean, median, and quantile distributions of the *Z*^2^ for both uncalibrated and calibrated TWAS results for each trait. Additionally, it has sample size, heritability, and category of each trait.

Code S1. The R code script used for simulation analysis

Document S2. Article plus supplemental information
